# The prognostic significance of CD11b^+^CX3CR1^+^ monocytes in patients with newly diagnosed diffuse large B-cell lymphoma

**DOI:** 10.18632/oncotarget.21241

**Published:** 2017-09-23

**Authors:** Ho-Young Yhim, Jeong-A Kim, Sun-Hye Ko, Youngrok Park, Eunjung Yim, Hee Sun Kim, Jae-Yong Kwak

**Affiliations:** ^1^ Department of Internal Medicine, Chonbuk National University Medical School, Jeonju, Republic of Korea; ^2^ Research Institute of Clinical Medicine, Chonbuk National University-Biomedical Research Institute of Chonbuk National University Hospital, Jeonju, Republic of Korea; ^3^ Division of Hematology, Department of Internal Medicine, College of Medicine, The Catholic University of Korea, Seoul, Republic of Korea; ^4^ Leukemia Research Institute, The Catholic University of Korea, Seoul, Republic of Korea; ^5^ Tumor Biology Training Program, Lombardi Comprehensive Cancer Center, Georgetown University School of Medicine, Washington, DC, USA; ^6^ College of Nursing, Chonbuk National University, Jeonju, Republic of Korea

**Keywords:** angiogenesis, CD11b, CX3CR1, diffuse large B-cell lymphoma, immunosuppression

## Abstract

Despite their critical roles in angiogenesis and host immunosuppression within the tumor microenvironment, the prognostic significance of myeloid-lineage cells expressing CD11b and CX3CR1 in diffuse large B-cell lymphoma (DLBCL) has not been well studied. We prospectively enrolled newly-diagnosed DLBCL patients at two Korean institutions between May 2011 and Aug 2015. CD11b^+^CX3CR1^+^ cells were analyzed by flow cytometry using peripheral blood (PB) and bone marrow (BM) aspirate samples before treatments. Eighty-nine patients (52 males) were enrolled. The median age was 65 years (range, 19–88 years). Thirty-seven patients (42%) were classified as high-intermediate or high risk according to the National Comprehensive Cancer Network International Prognostic Index (NCCN-IPI). Patients were categorized into either high or low PB-/BM-CD11b^+^CX3CR1^+^ monocyte group according to the cutoffs identified by the receiver-operating-characteristics analysis (PB, 3.68%; BM, 3.45%). The high PB-CD11b^+^CX3CR1^+^ monocyte group was significantly associated with high-intermediate and high risk NCCN-IPI group (*P* = 0.004). With a median follow-up of 27.7 months (range, 1.7-60.4 months), the low PB-CD11b^+^CX3CR1^+^ monocyte group showed significantly better overall survival (OS) than the high PB-CD11b^+^CX3CR1^+^ monocyte group (3-year, 92.3% *vs.* 51.2%, respectively; *P* < 0.001). In contrast, no significant difference was observed between the high and low BM-CD11b^+^CX3CR1^+^ monocyte groups. Among patients with high-intermediate to high risk NCCN-IPI, the high PB-CD11b^+^CX3CR1^+^ monocyte group showed significantly worse OS than the low PB-CD11b^+^CX3CR1^+^ monocyte group (3-year, 29.3% *vs.* 80.2%, respectively; *P* = 0.008). Taken together, PB-CD11b^+^CX3CR1^+^ monocyte percentage correlates with the NCCN-IPI risk stratification, which enables identification of subgroups with extremely poor clinical outcomes.

## INTRODUCTION

Diffuse large B-cell lymphoma (DLBCL) is the most common histological subtype of aggressive non-Hodgkin's lymphomas in Korea [1]. Although DLBCL is classified as a single disease, it encompasses a wide variety of tumor biology and therefore results in different clinical behaviors, responses to therapy, and long-term outcomes [2]. Although the addition of rituximab to conventional therapy with cyclophosphamide, doxorubicin, vincristine, and prednisone (R-CHOP) has vastly improved clinical outcomes, a significant number of patients still experience treatment failure and eventually die [2]. Therefore, an improved risk stratification method and novel treatment approaches in groups at high risk of treatment failure are greatly needed.

Myeloid-lineage cells, including monocytes and their precursors, play central roles in cancer growth and progression by directly promoting angiogenesis [3, 4] and suppressing anti-tumor immunity [5–8]. Recently, interest in the prognostic role of myeloid-lineage cells in lymphoma has been increasing [7, 9–11]. Several investigators have reported that high circulating monocyte counts in combination with low lymphocyte counts have prognostic relevance in patients with DLBCL treated with R-CHOP, particularly high-risk DLBCL patients [10–12]. These findings suggest that specific subsets of monocytes may be responsible for cancer prognosis in DLBCL.

Among the various subsets of monocytes, cells expressing CD11b and CX3CR1 have been shown to promote angiogenesis through interactions with fractalkine (CX3CL1), the only ligand for CX3CR1, in mouse ischemic models [13]. CD11b^+^CX3CR1^+^ monocytes are a subpopulation of CD11b^+^Gr1^+^ cells [14] that were shown to be myeloid-derived suppressor cells (MDSCs) in mouse tumor models [6]. Based on suppression of host anti-tumor immunity mediated by CD11b^+^CX3CR1^+^ monocytes in solid cancer models [15–17] and the upregulation of CX3CR1 receptor in various types of B-cell lymphoma including DLBCL [18], we hypothesized that CD11b^+^CX3CR1^+^ monocytes might influence the prognosis of patients with DLBCL by promoting angiogenesis and immunosuppression within the tumor microenvironment. However, there has been no data on the prognostic relevance of CD11b^+^CX3CR1^+^ monocytes in DLBCL as well as its relationship with clinical variables. Therefore, this prospective study investigated the prognostic significance of CD11b^+^CX3CR1^+^ monocytes in peripheral blood (PB) and bone marrow (BM) on survival outcomes in newly-diagnosed DLBCL patients treated with R-CHOP immunochemotherapy.

## RESULTS

### Patient cohort and characteristics

**Figure 1:**
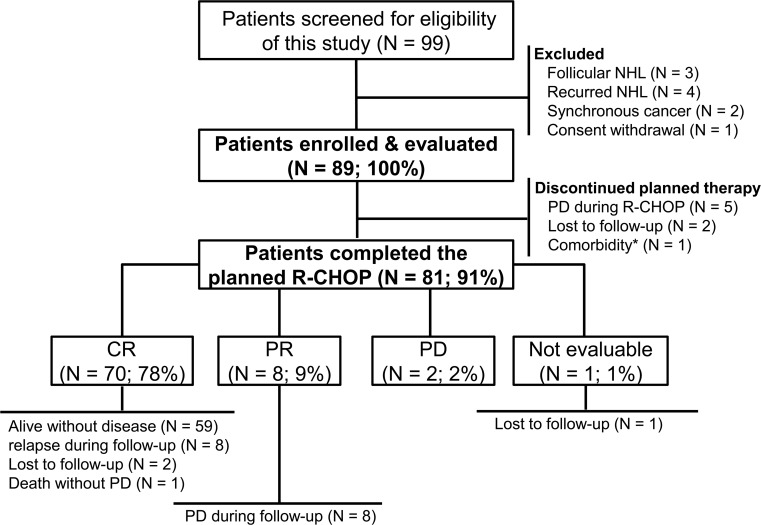
Flowchart of the study NHL, non-Hodgkin lymphoma; R-CHOP, rituximab plus cyclophosphamide, doxorubicin, vincristine, and prednisone; PD, progressive disease; CR, complete response; PR, partial response.^*^Femur intertrochanteric fracture following a fall by a 79-year-old woman.

Ninety-nine patients were screened for this study. Ten patients were excluded; nine patients did not meet the eligibility criteria, and one patient withdrew informed consent. A total of 89 patients were enrolled in the study, and their data were analyzed (Figure 1). The median age was 65 years at diagnosis (range, 19–88 years), and 52 patients (58.4%) were male. Approximately half of the patients had advanced stage disease (N = 45, 50.6%) and elevated serum lactate dehydrogenase (LDH) levels (N = 46, 51.7%; Table 1). Fifty-two patients (58.4%) were classified as non-germinal center B-cell like phenotype by immunohistochemistry [19]. Thirty-seven patients (41.6%) were classified as high-intermediate or high risk based on the National Comprehensive Cancer Network-International Prognostic Index (NCCN-IPI).

**Table 1:** Clinical characteristics and the association with PFS and OS

Variables	No. (%)	PFS				OS			
		Univariate analysis		Multivariate analysis		Univariate analysis		Multivariate analysis	
		3-year (%, 95% CI)	*P*	HR (95% CI)	*P*	3-year (%, 95% CI)	*P*	HR (95% CI)	*P*
Age (years) <60 ≥60	38 (42.7) 51 (57.3)	75.3 (57.7-92.9) 63.5 (47.8-79.2)	0.218			89.5 (78.3-100.0) 66.7 (51.4-82.0)	0.045		
Sex Male Female	52 (58.4) 37 (41.6)	71.4 (56.7-86.1) 65.7 (47.3-84.1)	0.488			81.4 (69.4-93.4) 69.2 (51.0-87.4)	0.551		
Ann Arbor stage I to II III to IV	44 (49.4) 45 (50.6)	74.5 (58.8-90.2) 64.1 (44.7-79.5)	0.280			86.1 (74.5-97.7) 66.6 (50.1-83.1)	0.083		
Performance status ECOG 0/1 ECOG ≥2	74 (83.1) 15 (16.9)	78.8 (67.2-90.4) 28.0 (2.9-53.1)	<0.001			86.5 (77.1-95.9) 37.3 (11.2-63.4)	<0.001		
Serum LDH level Normal Elevated	43 (48.3) 46 (51.7)	69.1 (53.0-85.2) 69.3 (52.6-86.0)	0.886			78.1 (64.6-91.6) 75.8 (60.7-90.9)	0.766		
B symptoms Absence Presence	59 (66.3) 30 (33.7)	74.7 (61.6-87.8) 57.6 (35.8-79.4)	0.202			85.0 (74.6-95.4) 60.4 (39.6-81.2)	0.026		
Bulky disease No Yes	80 (89.9) 9 (10.1)	68.2 (55.7-80.7) 71.1 (35.8-100.0)	0.890			77.3 (66.7-87.9) 71.1 (35.8-100.0)	0.818		
Extranodal involvement No Yes	34 (38.2) 55 (61.8)	86.1 (73.4-98.8) 58.5 (42.4-74.6)	0.031			88.7 (76.4-100.0) 69.7 (55.6-83.8)	0.045		
Cell of origin GCB phenotype Non-GCB phenotype Unknown	35 (39.3) 52 (58.4) 2 (2.3)	73.2 (56.5-89.9) 65.2 (48.9-81.5) 100.0	0.482			75.6 (59.7-91.5) 77.4 (64.1-90.7) 100.0	0.900		
NCCN-IPI Low/low-intermediate High-intermediate/high	52 (58.4) 37 (41.6)	83.6 (70.5-96.7) 47.3 (27.7-66.9)	<0.001	3.67 (1.40-9.62)	0.008	95.1 (88.4-100.0) 51.3 (32.1-70.5)	<0.001	16.25 (1.76-22.18)	0.005
PB-CD11b^+^CX3CR1^+^ cells Low High	52 (58.4) 37 (41.6)	80.0 (65.7-94.3) 51.6 (42.1-61.1)	<0.001			92.3 (83.9-100.0) 51.2 (31.2-71.2)	<0.001		
BM-CD11b^+^CX3CR1^+^ cells Low High	50 (56.8) 38 (43.2)	75.3 (59.4-91.2) 60.1 (42.7-77.5)	0.193			81.3 (68.6-94.0) 71.3 (55.0-87.6)	0.355		
PB-/BM-CD11b^+^CX3CR1^+^ cells ratio ≤1.77 >1.77	61 (69.3) 27 (30.7)	70.7 (56.0-85.4) 63.6 (44.2-83.0)	0.118			78.4 (66.2-90.6) 72.9 (54.3-91.5)	0.811		

Abbreviations: PFS, progression-free survival; OS, overall survival; CI, confidence interval; ECOG, Eastern Cooperative Oncology Group; LDH, lactate dehydrogenase; GCB, germinal center B-cell like; NCCN-IPI, National Comprehensive Cancer Network-International Prognostic Index (NCCN-IPI); PB, peripheral blood; BM, bone marrow.

### Baseline PB- and BM-CD11b^+^CX3CR1^+^ monocytes and survival outcomes

**Figure 2:**
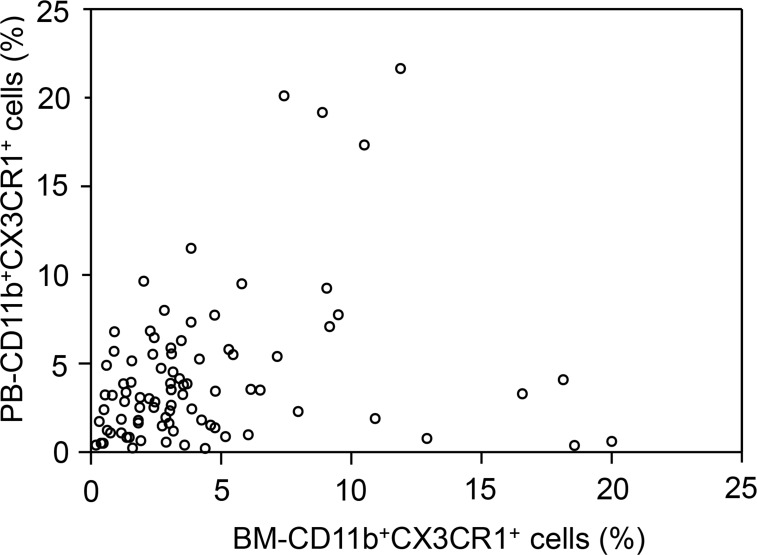
Scatter plot of the percentages of bone marrow (BM)-CD11b^+^CX3CR1^+^ versus peripheral (PB)-CD11b^+^CX3CR1^+^ monocytes in each patient A dot was not marked on this plot for one patient because BM-CD11b^+^CX3CR1^+^ monocyte percentage was not available. There was no association between the percentages of PB- and BM-CD11b^+^CX3CR1^+^ monocytes (Spearman correlation coefficient = 0.202; *P* = 0.132).

Flow cytometric analyses of PB samples were successfully performed in all 89 patients. However, BM aspirate samples were analyzed in 88 patients, because one of the samples was not appropriate for analysis. CD11b^+^CX3CR1^+^ monocytes were detectable in all PB and BM aspirate samples analyzed. The median percentages of CD11b^+^CX3CR1^+^ monocytes were 3.31% (range, 0.21–21.66%) in PB (PB-CD11b^+^CX3CR1^+^ monocytes) and 3.09% (range, 0.20–20.01%) in BM (BM-CD11b^+^CX3CR1^+^ monocytes). There was no association between the percentages of PB- and BM-CD11b^+^CX3CR1^+^ cells (Spearman correlation = 0.202, *P* = 0.132; Figure 2).

**Figure 3:**
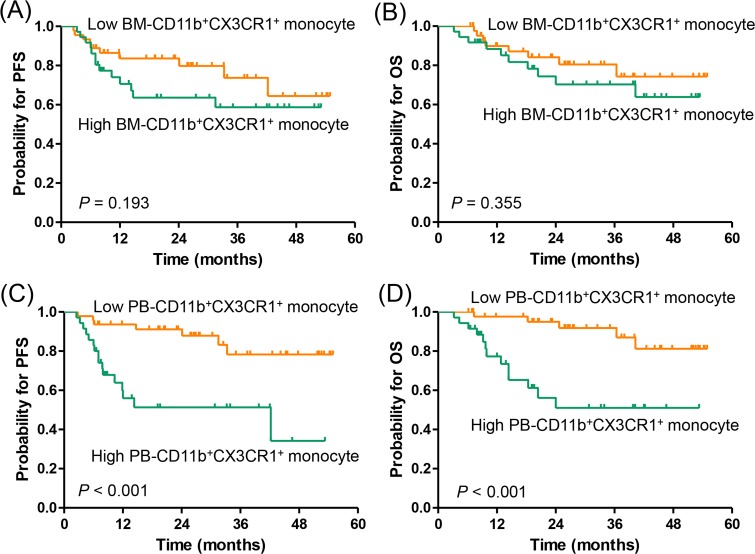
Progression-free survival and overall survival according to CD11b^+^CX3CR1^+^ monocyte percentage **(A, B)** bone marrow aspirate and **(C, D)** peripheral blood samples.PFS, progression-free survival; OS, overall survival; BM, bone marrow; PB, peripheral blood.

Responses to R-CHOP therapy were evaluable in 85 (95.5%) of the 89 patients (Supplementary Table 1). Among the evaluable patients, 70 patients (82.4%) achieved a complete response (CR), which was significantly higher in patients with low or low-intermediate NCCN-IPI (46/49 [93.9%] *vs.* 24/36 [66.7%]; *P* = 0.001) and low PB-CD11b^+^CX3CR1^+^ monocyte group (47/51 [92.2%] *vs.* 23/34 [66.7%]; *P* = 0.004). During the median follow-up of 27.7 months (range, 1.7-60.4 months), 23 patients relapsed or progressed, and 18 patients died, including 1 patient with non-disease-related death. The estimated 3-year progression-free survival (PFS) and overall survival (OS) rates were 68.7% (95% confidence interval [CI], 56.9–80.5) and 76.8% (95% CI, 66.6–87.0), respectively. There were no significant differences in PFS (*P* = 0.193) or OS (*P* = 0.355) between the high and low BM-CD11b^+^CX3CR1^+^ monocyte groups (Figure 3A, 3B). In contrast, the high PB-CD11b^+^CX3CR1^+^ monocyte group had more patients with relapse/progression (16/37 [43.2%] *vs.* 7/52 [13.5%]) and death (13/37 [35.1%] *vs.* 5/52 [9.6%]) than the low PB-CD11b^+^CX3CR1^+^ monocyte group. Thus, high PB-CD11b^+^CX3CR1^+^ monocyte group was significantly associated with worse PFS (3-year, 51.6% vs. 80.0%; *P* < 0.001) and OS (3-year, 51.2% vs. 92.3%; *P* < 0.001; Figure 3C, 3D).

Univariate analyses of PFS and OS demonstrated that Eastern Cooperative Oncology Group (ECOG) performance status, extranodal involvement, risk stratification by NCCN-IPI (Supplementary Figure 1), and PB-CD11b^+^CX3CR1^+^ cell groups were significantly associated with PFS and OS (Table 1). Age ≥60 years and the presence of B symptoms were also associated with increased death. However, high-intermediate to high risk NCCN-IPI was the only independent prognostic factor for shorter PFS (hazard ratio [HR], 3.67; 95% CI, 1.40–9.62) and OS (HR, 6.25; 95% CI, 1.76–22.18) in multivariate analysis (Table 1).

### Association between baseline PB- and BM-CD11b^+^CX3CR1^+^ monocytes and other clinical variables

We investigated the association of CD11b^+^CX3CR1^+^ monocyte percentages in PB or BM with other clinical variables. The high PB-CD11b^+^CX3CR1^+^ monocyte group was significantly associated with unfavorable clinical variables, including ECOG performance status ≥2 (*P* = 0.031), elevated serum LDH levels (*P* = 0.036), and extranodal involvement (*P* = 0.003), and also showed a trend towards older age (*P* = 0.099) (Table 2). In particular, PB-CD11b^+^CX3CR1^+^ monocyte percentages were significantly associated with risk stratification by NCCN-IPI (*P* = 0.004). However, BM-CD11b^+^CX3CR1^+^ monocyte percentages were not significantly associated with any other clinical variables (Table 2).

**Table 2:** The association between baseline CD11b^+^CX3CR1^+^ cells and clinical variables

Variables	PB-CD11b^+^CX3CR1^+^ cells(N = 89)				BM-CD11b^+^CX3CR1^+^ cells(N = 88)			
	No. (%)	Low (N = 52, %)	High (N = 37, %)	*P*	No. (%)	Low (N = 50, %)	High (N = 38, %)	*P*
Age (years) <60 ≥60	38 (42.7)51 (57.3)	26 (50.0)26 (50.0)	12 (32.4)25 (67.6)	0.099	38 (43.2)50 (56.8)	23 (46.0)27 (54.0)	15 (39.5)23 (60.5)	0.540
Sex Male Female	52 (58.4)37 (41.6)	32 (61.5)20 (38.5)	20 (54.1)17 (45.9)	0.480	51 (58.0)37 (42.0)	27 (54.0)23 (46.0)	24 (63.2)14 (36.8)	0.389
Ann Arbor stage I to II III to IV	44 (49.4)45 (50.6)	28 (53.8)24 (46.2)	16 (43.2)21 (56.8)	0.324	44 (50.0)44 (50.0)	25 (50.0)25 (50.0)	19 (50.0)19 (50.0)	1.000
Performance status ECOG 0/1 ECOG ≥2	74 (83.1)15 (16.9)	47 (90.4)5 (9.6)	27 (73.0)10 (27.0)	0.031	73 (83.0)15 (17.0)	44 (88.0)6 (12.0)	29 (76.3)9 (23.7)	0.149
Serum LDH level Normal Elevated	43 (48.3)46 (51.7)	30 (57.7)22 (42.3)	13 (35.1)24 (64.9)	0.036	43 (48.9)45 (51.1)	25 (50.0)25 (50.0)	18 (47.4)20 (52.6)	0.807
B symptoms Absence Presence	59 (66.3) 30 (33.7)	36 (69.2) 16 (30.8)	23 (62.2) 14 (37.8)	0.487	58 (65.9) 30 (34.1)	36 (72.0) 14 (28.0)	22 (57.9) 16 (42.1)	0.167
Bulky disease No Yes	80 (89.9) 9 (10.1)	47 (90.4) 5 (9.6)	33 (89.2) 4 (10.8)	0.854	79 (89.8) 9 (10.2)	46 (92.0) 4 (8.0)	33 (86.8) 5 (13.2)	0.429
Extranodal involvement No Yes	34 (38.2) 55 (61.8)	25 (48.1) 27 (51.9)	9 (24.3) 28 (75.7)	0.023	34 (38.2) 54 (61.8)	22 (44.0) 28 (56.0)	12 (31.6) 26 (68.4)	0.236
Cell of origin GCB Non-GCB	87 (100.0) 35 (40.2) 52 (59.8)	22 (43.1) 29 (56.9)	13 (36.1) 23 (63.9)	0.510	86 (100.0) 35 (40.7) 51 (59.3)	20 (41.7) 28 (58.3)	15 (39.5) 23 (60.5)	0.837
NCCN-IPI Low/low-intermediate High-intermediate/high	52 (58.4) 37 (41.6)	37 (71.2) 15 (28.8)	15 (40.5) 22 (59.5)	0.004	52 (59.1) 36 (40.9)	32 (64.0) 18 (36.0)	20 (52.6) 18 (47.4)	0.283

Abbreviations: BM, bone marrow; ECOG, Eastern Cooperative Oncology Group; LDH, lactate dehydrogenase; GCB, germinal center B-cell like; NCCN-IPI, National Comprehensive Cancer Network-International Prognostic Index.

**Figure 4:**
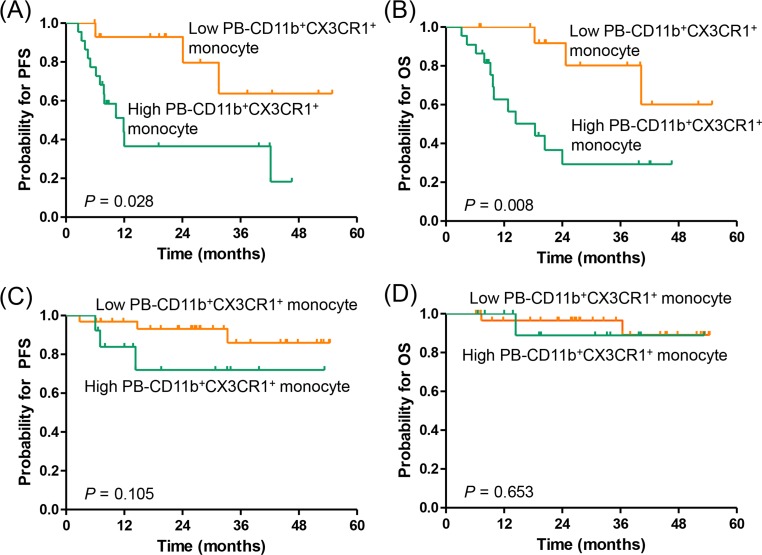
Subgroup analysis for the association of PB-CD11b^+^CX3CR1^+^ monocytes with progression-free survival and overall survival based on NCCN-IPI risk **(A, B)** high-intermediate to high risk NCCN-IPI (N = 37), **(C, D)** low to low-intermediate risk NCCN-IPI (N = 52). PFS, progression-free survival; OS, overall survival; BM, bone marrow; PB, peripheral blood.

Next, we classified the patients according to NCCN-IPI risk and evaluated the impact of PB-CD11b^+^CX3CR1^+^ monocytes on survival outcomes in each subgroup. Thirty-seven patients (41.6%) were classified as higher risk (high-intermediate to high) subgroup and 52 patients (58.4%) were classified as lower risk (low to low-intermediate) subgroups based on the NCCN-IPI. In the higher risk NCCN-IPI subgroup, patients with a high percentage of PB-CD11b^+^CX3CR1^+^ monocytes had significantly worse PFS (3-year, 36.5% *vs.* 59.4%, respectively; *P* = 0.028) and OS (3-year, 29.3% *vs.* 80.2%, respectively; *P* = 0.008) than those with a low percentage of PB-CD11b^+^CX3CR1^+^ monocytes (Figure 4A, 4B). However, in the lower risk NCCN-IPI subgroup, the PB-CD11b^+^CX3CR1^+^ monocyte percentages failed to predict PFS and OS (Figure 4C, 4D).

## DISCUSSION

In this prospective cohort study involving 89 DLBCL patients, we investigated the impact of PB- and BM-CD11b^+^CX3CR1^+^ monocytes on survival outcomes in patients with newly diagnosed DLBCL treated with R-CHOP immunochemotherapy. Our study demonstrated that the patients with high percentage of PB-CD11b^+^CX3CR1^+^ monocytes were significantly associated with disease progression and death, compared to those with low percentage of PB-CD11b^+^CX3CR1^+^ monocytes. A high percentage of PB-CD11b^+^CX3CR1^+^ monocytes was also strongly associated with several poor prognostic variables, including poor ECOG performance status, elevated serum LDH levels, extranodal involvement, and higher NCCN-IPI risk. Furthermore, the baseline percentage of PB-CD11b^+^CX3CR1^+^ monocytes allowed us to identify the subgroup with very poor outcomes among patients with higher risk NCCN-IPI.

Prior to the present study, there were no data regarding the prognostic value of CD11b^+^CX3CR1^+^ monocytes in DLBCL. As we hypothesized, a high percentage of PB-CD11b^+^CX3CR1^+^ monocytes was clearly associated with disease progression and death. The significant role of PB-CD11b^+^CX3CR1^+^ monocytes on PFS and OS in patients with newly diagnosed DLBCL might be attributed to the pro-angiogenic capacity of these cells. In the tumor microenvironment, myeloid-lineage cells play important roles in promoting tumor angiogenesis [3, 4]. Yang *et al.* [4] reported that myeloid-lineage cells expressing CD11b and Gr1 directly contributed to tumor angiogenesis. However, CD11b^+^Gr1^+^ cells are heterogeneous, containing various subpopulations, such as CD11b^+^CX3CR1^+^, CD11b^+^CCR2^+^, CD11b^+^CCR5^+^, and CD11b^+^CXCR4^+^ cells [14]. Therefore, it is necessary to define the specific subset that enhances tumor progression. Recently, CD11b^+^CX3CR1^+^ monocytes have been found to strongly promote endothelial proliferation in human umbilical vein endothelial cell culture through CX3CR1/fractalkine interaction [13]. Although the pro-angiogenic effect of CD11b^+^CX3CR1^+^ monocytes has not been well described in human cancer types, these findings suggest that CD11b^+^CX3CR1^+^ monocytes might promote tumor growth and progression by enhancing angiogenesis in DLBCL.

In addition to their pro-angiogenic capacity, PB-CD11b^+^CX3CR1^+^ monocytes can suppress host anti-tumor immune responses. In a mouse model of ovarian cancer, CX3CR1-expressing myeloid cells promoted immunosuppression through inhibition of T-cell activity by IL-10 [15, 20], suggesting that CD11b^+^CX3CR1^+^ cells function as MDSCs. In this study, we identified the subset of CD11b^+^ myelo-monocytic cells expressing CX3CR1 in the PB and BM of DLBCL patients. Since these cells were detected in both mice and humans [14], we postulated that they promote the immunosuppressive tumor microenvironment in DLBCL patients through immunosuppressive mechanisms similar to those observed in mice. Therefore, although previous studies have suggested the immunosuppressive role of CD11b^+^CX3CR1^+^ monocytes in the tumor microenvironment, the mechanism of these cells in DLBCL needs to be further clarified.

In contrast to PB-CD11b^+^CX3CR1^+^ monocytes, BM-CD11b^+^CX3CR1^+^ monocytes were not associated with prognosis of DLBCL patients. It is unclear why only PB-CD11b^+^CX3CR^+^ monocytes affect the prognosis of DLBCL patients. Considering that BM-derived myeloid-lineage cells are generally under steady state conditions, our findings suggest that additional stimulants may be required to mobilize these cells from the BM into the PB [21]. Fractalkine, a unique member of the CX3C family, is a transmembrane protein that can be cleaved into a soluble form with potent chemoattractant properties for monocytes [22, 23]. Truman L *et al.* [24] reported that apoptotic lymphoma cells released fractalkine and recruited CX3CR1-expressing monocytes to the germinal center in a mouse model of lymphoma. Therefore, we postulate that tumor-derived fractalkine release stimulates mobilization of CD11b^+^CX3CR1^+^ monocytes through fractalkine/CX3CR1 interaction, and these mobilized CD11b^+^CX3CR1^+^ monocytes in the PB have angiogenic and/or immunosuppressive activity in the tumor microenvironment.

Our study also demonstrated that PB-CD11b^+^CX3CR1^+^ monocytes were strongly correlated with the risk stratification by NCCN-IPI, demonstrating the association of PB-CD11b^+^CX3CR1^+^ monocytes with clinical variables in DLBCL. NCCN-IPI is a new prognostic tool in the era of rituximab-based therapy, which incorporates a refined categorization of age, serum LDH level, and disease involvement at specific extranodal sites (i.e., BM, central nervous system, liver, gastrointestinal tract, or lung) [25]. With regards to age and serum LDH level, NCCN-IPI shows more detailed values; therefore, it is able to discriminate high risk groups better than the original IPI [25]. In addition to the enhanced capacity of NCCN-IPI for predicting prognosis in DLBCL, the results of our study revealed that PB-CD11b^+^CX3CR1^+^ monocyte level might be used to further discriminate the risk of disease progression and death in higher risk NCCN-IPI subgroup. Thus, our results will be useful for discriminating patients at high risk for disease progression and death. Furthermore, these findings have therapeutic implications. For lower risk NCCN-IPI subgroup, traditional R-CHOP chemotherapy shows excellent outcomes regardless of PB-CD11b^+^CX3CR1^+^ monocyte status. In contrast, patients in the higher risk NCCN-IPI subgroup with high PB-CD11b^+^CX3CR1^+^ monocyte percentages showed very poor OS (3-year, 29%). Interestingly, the lower survival rate observed in this study may be improved by adding novel drugs that inhibit fractalkine/CX3CR1 signaling. A novel small-molecule CX3CR1 antagonist has shown preclinical evidence of activity by interfering with progression and metastasis in a breast cancer model [26]. Thus, clinical application of new therapeutic agents that target the fractalkine/CX3CR1 axis could be applied in the high risk DLBCL patients with high percentage of PB-CD11b^+^CX3CR1^+^ monocytes.

A potential limitation of our study is the relatively small number of patients with short follow-up period (approximately 2.3 years). Indeed, only approximately 20% of patients died, reflecting the limitation of an arbitrary cutoff rather than the biological cutoff that reflects the different clinical outcomes between the high and low CD11b^+^CX3CR1^+^ monocyte groups. Furthermore, although our subgroup analysis provided some useful information, the results of the subgroup analysis might be underpowered. Future studies with larger populations are required to bolster our results.

Taken together, our study establishes the significant association of PB-CD11b^+^CX3CR1^+^ monocytes with risk stratification with the NCCN-IPI, and also identifies a subgroup with extremely poor clinical outcomes. This study also suggests the possibility of PB-CD11b^+^CX3CR1^+^ monocytes as a potential therapeutic target in higher risk subgroup of DLBCL.

## MATERIALS AND METHODS

### Study design and eligibility criteria

From May 2011 to August 2015, this multicenter, prospective, pilot study included patients with newly diagnosed, biopsy-proven, CD20-positive DLBCL. Other eligibility criteria included no prior history of chemotherapy or radiotherapy and adequate organ function to receive chemotherapy with curative intent. Patients were excluded if they had secondary transformed DLBCL, symptomatic central nervous system involvement, or other concurrent cancers requiring treatments. Patients suffering from severe infection, liver cirrhosis, or psychiatric disorder were also excluded. This study was approved by the Institutional Review Board at each institution. All patients provided written informed consent.

### Evaluation and treatment

Patients were staged according to the Ann Arbor staging system. Baseline staging evaluation included a physical examination, complete blood count, serum biochemistry with LDH, computed tomographic (CT) scanning of the chest and abdomen-pelvis, CT scanning or magnetic resonance imaging of other involved lesions, fluorodeoxyglucose positron emission tomography, and bilateral BM aspiration and trephine biopsy. Predefined treatment consisted of six to eight cycles of standard R-CHOP immunochemotherapy (rituximab 375 mg/m^2^, cyclophosphamide 750 mg/m^2^, doxorubicin 50 mg/m^2^, and vincristine 1.4 mg/m^2^ intravenously one day 1, and prednisone 100mg orally for 5 days) every 21 days. Prophylactic granulocyte-colony stimulating factor was recommended but was not mandated. Dose modification with respect to adverse effects in the preceding cycle was performed at the discretion of treating physician. The NCCN-IPI was determined for prognosis [25]. Responses to treatment were assessed by modified response criteria [27].

### Measurements of baseline CD11b^+^CX3CR1^+^ monocytes in PB and BM aspirate samples

**Figure 5:**
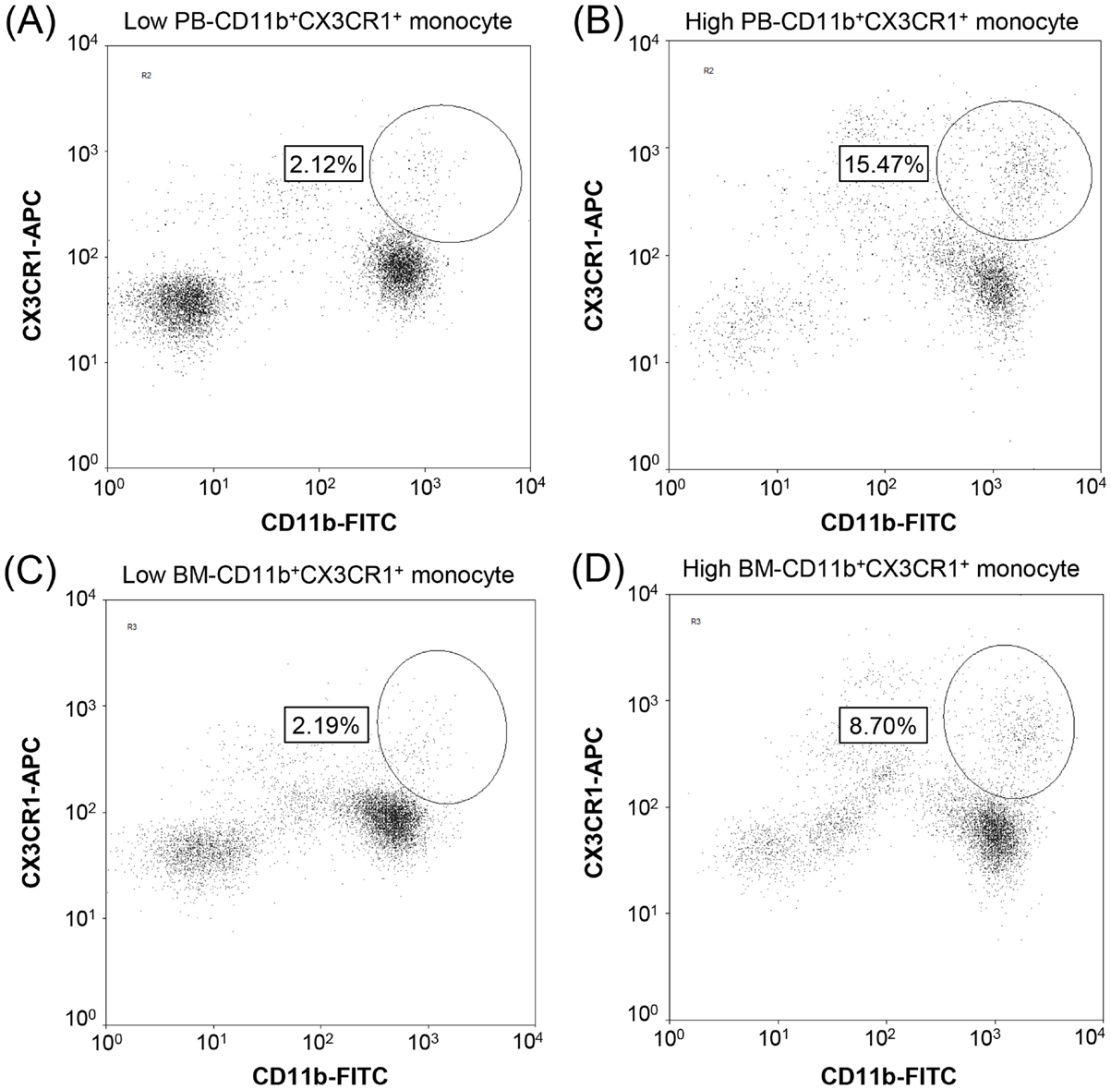
Identification of CD11b^+^CX3CR1^+^ monocytes in clinical samples Representative flow cytometric analysis results showing the percentages of CD11b^+^CX3CR1^+^ monocytes in peripheral blood **(A, B)** and bone marrow **(C, D)** aspirate samples, which were measured as the percentage of CD11b^+^CX3CR1^+^ monocytes among total mononuclear cells (> 50,000 cells). Patients were categorized into low (A, C) or high (B, D) PB- and BM-CD11b^+^CX3CR1^+^ monocyte groups.

The percentage of CD11b^+^CX3CR1^+^ cells among the total mononuclear cells (MNCs) was measured by flow cytometric analysis in fresh PB and BM aspirate samples before administration of R-CHOP therapy. PB- and BM-CD11b^+^CX3CR1^+^ cells were measured by the same procedure. Briefly, 2 mL of PB or BM aspirates was added to 20 mL of RBC lysis buffer (1×, Biolegend, San Diego, CA), and then the cells were stained with APC-conjugated anti-human CX3CR1 antibody (Ab; Biolegend, San Diego, CA; catalog number 2A9-1), and FITC-conjugated anti-human CD11b Ab (eBioscience, San Diego, CA; catalog number 11-0118) for 30 min at 4°C. At least 50,000 MNCs were gated based on forward- and side-scatter profiles, and further subdivided into CD11b^+^ and CX3CR1^+^ subpopulations. The percentage of CD11b^+^CX3CR1^+^ cells was analyzed using a Navios flow cytometer (Beckman Coulter, Brea, CA; Figure 5).

### Statistical analysis

Pearson's χ^2^ test or Fisher's exact test was used to analyze the association between the baseline CD11b^+^CX3CR1^+^ monocyte percentages and clinical variables, such as age, sex, Ann Arbor stage, serum LDH level, the ECOG performance status, presence of B symptoms, bulky disease, extranodal involvement, cell of origin by Hans classification [19], and the NCCN-IPI [25]. To determine the optimal cutoffs for PB- and BM-CD11b^+^CX3CR1^+^ cells and the ratio of PB/BM-CD11b^+^CX3CR1^+^ cells for predicting disease progression or death, receiver-operating-characteristics (ROC) curve analysis was used. ROC curve analysis demonstrated that the cutoffs of 3.68% (PB), 3.45% (BM), and 1.77 (PB/BM) had the best sensitivity and specificity to predict disease progression or death. Then, patients were categorized into high or low PB- or BM-CD11b^+^CX3CR1^+^ monocyte groups according to the cutoffs. Spearman's correlation coefficient was used to assess the association between PB-CD11b^+^CX3CR1^+^ and BM-CD11b^+^CX3CR1^+^ cells. PFS and OS were defined as the time from the date of diagnosis to the date of disease progression, death, or last follow-up, as appropriate, and were estimated using the Kaplan-Meier method. Differences in survival outcomes between two groups were tested using the log-rank test. A Cox proportional hazards regression model was used for univariate and multivariate analysis of PFS and OS. Variables with *P* <0.05 in the univariate analysis were included in the multivariate analysis; a forward conditional method was used, and the results were reported as a hazard ratio and 95% CI. A two-sided *P* <0.05 was considered statistically significant. All data analyses were carried out using SPSS software, version 19.0 (SPSS Inc., Chicago, IL).

## SUPPLEMENTARY MATERIALS FIGURE AND TABLE



## Abbreviations

BM: Bone marrow
CR: complete response
CT: computed tomography
DLBCL: diffuse large B-cell lymphoma
LDH: lactate dehydrogenase
MNC: mononuclear cell
MDSCs: myeloid-derived suppressor cells
NCCN-IPI: National Comprehensive Cancer Network-International Prognostic Index
OS: overall survival
PB: peripheral blood
PFS: progression-free survival
ROC: receiver-operating-characteristics
R-CHOP: Rituximab with cyclophosphamide, doxorubicin, vincristine, and prednisone
