# Supplementary material for: The prognostic significance of CD11b+CX3CR1+ monocytes in patients with newly diagnosed diffuse large B-cell lymphoma
Source: Oncotarget. 2017 Sep 23;8(54):92289–99. doi: 10.18632/oncotarget.21241 (PMC5696181; doi:10.18632/oncotarget.21241)
Supplement: Supplementary file 1 [file oncotarget-08-92289-s001.pdf]

The prognostic significance of CD11b$^+$CX3CR1$^+$ monocytes in patients with newly diagnosed diffuse large B-cell lymphoma

SUPPLEMENTARY MATERIALS

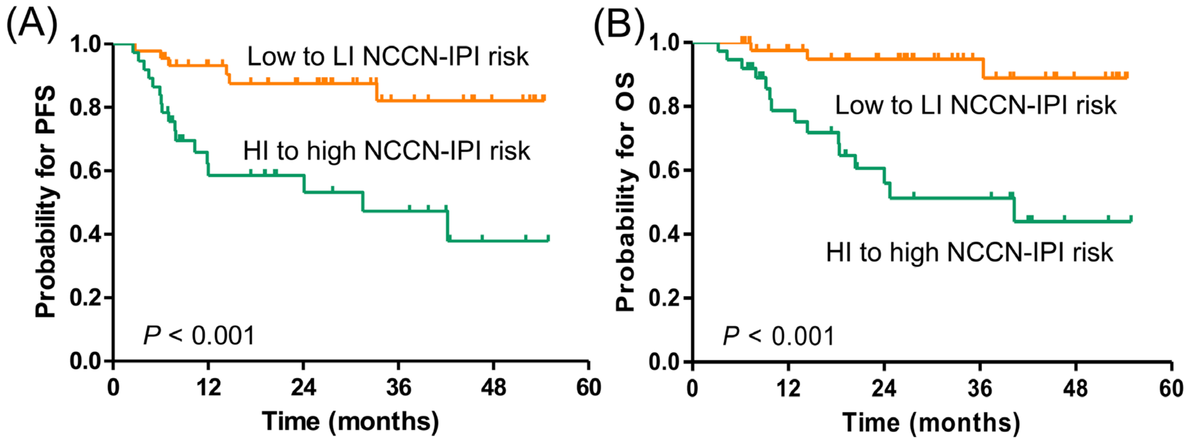

Supplementary Figure 1: Progression-free survival and overall survival according to the NCCN-IPI risk. **(A)** PFS, **(B)** OS PFS, progression-free survival; OS, overall survival; NCCN-IPI, National Comprehensive Cancer Network International Prognostic Index; LI, low-intermediate; HI, high-intermediate.

Supplementary Table 1: Treatment response to immunochemotherapy according to the baseline clinical variables

	Patients (N = 85)	CR	Non-CR	*P*
Age (years)				
<60	38	33 (86.8)	5 (13.2)	0.329
≥60	47	37 (78.7)	10 (21.3)	
Sex				
Male	50	41 (82.0)	9 (18.0)	0.919
Female	35	29 (82.9)	6 (17.1)	
Ann Arbor stage				
I to II	43	36 (83.7)	7 (16.3)	0.738
III to IV	42	34 (81.0)	8 (19.0)	
Performance status				
ECOG 0/1	71	62 (87.3)	9 (12.7)	0.015
ECOG ≥2	14	8 (57.1)	6 (42.9)	
Serum LDH level				
Normal	41	34 (82.9)	7 (17.1)	0.893
Elevated	44	36 (81.8)	8 (18.2)	
B symptoms				
Absence	55	47 (85.5)	8 (14.5)	0.310
Presence	30	23 (76.7)	7 (23.3)	
Bulky disease				
No	76	63 (82.9)	13 (17.1)	0.656
Yes	9	7 (77.8)	2 (22.2)	
Extranodal involvement				
No	32	29 (90.6)	3 (9.4)	0.120
Yes	53	41 (77.4)	12 (22.6)	
Cell of origin				
GCB	34	29 (85.3)	5 (14.7)	0.534
Non-GCB	50	40 (80.0)	10 (20.0)	
NCCN-IPI				
Low/low-intermediate	49	46 (93.9)	3 (6.1)	0.001
High-intermediate/high	36	24 (66.7)	12 (33.3)	
PB-$CD11b^+CX3CR1^+$ cells				
Low	51	47 (92.2)	4 (7.8)	0.004
High	34	23 (67.6)	11 (32.4)	
BM-$CD11b^+CX3CR1^+$ cells				
Low	47	40 (85.1)	7 (14.9)	0.424
High	37	29 (78.4)	8 (21.6)	

Abbreviations: CR, complete response; ECOG, Eastern Cooperative Oncology Group; LDH, lactate dehydrogenase; GCB, germinal center B-cell; NCCN-IPI, National Comprehensive Cancer Network-International Prognostic Index; PB, peripheral blood; BM, bone marrow.
